# The perils of conducting meta-analyses of observational data

**DOI:** 10.7448/IAS.17.1.19112

**Published:** 2014-06-09

**Authors:** Guy Harling, SV Subramanian

**Affiliations:** 1 Department of Global Health and Population, Harvard School of Public Health, Boston, MA, USA; 2 Department of Social and Behavioral Sciences, Harvard School of Public Health, Boston, MA, USA

Dear Editors,

We read with interest Li and colleagues’ recent article in this journal [1], which conducted a systematic review and meta-analysis of the literature on associations between intimate partner violence (IPV) and HIV infection in women. The authors found a significant positive association between various types of IPV and HIV, concluding that “physical violence, sexual violence, a combination of physical and sexual violence, and any type of IPV were associated with HIV infection in women (p. 7)” [1].

The authors noted in their Table 1 that a past study of which we were co-authors reported no significant association between IPV and HIV in 10 Demographic and Health Surveys conducted between 2003 and 2007 [2]. We were therefore surprised to see that, in conducting their meta-analysis, the authors report that our data show significant positive relationships between HIV and several types of IPV (Figures 2 and 3 in [1]). With the exception of the figure for “any type of violence” which appears to be a typographical error, the figures quoted seem congruent with the unadjusted, pooled numbers of individuals in four groups – exposed and unexposed, with and without HIV – across all countries, as provided in Table 1 of our paper [2]. In seeking to understand this discrepancy between the interpretations of our data made by ourselves and Li and colleagues, we have come to the conclusion that, while the authors have followed many of the recommendations of the Meta-analysis of Observational Studies in Epidemiology (MOOSE) Group [3], certain aspects of their analysis lead to concern regarding the findings they present.

First, in integrating two multi-country studies [2, 4], Li and colleagues have hidden any heterogeneity present within studies, by aggregating together data from all countries without allowing for country-level confounding. As can be seen in Table 3 of our paper [2], simply adding a fixed-effect indicator for each country into a bivariate logistic model with a binary exposure considerably attenuates the association between IPV and HIV. This change reflects confounding and effect-modification at the country level due to a positive relationship between country-level IPV rates and country-level HIV rates in our sample. Li and colleagues could have avoided this forced homogeneity by using country-specific numbers for each of the four exposure/outcome categories: when we analyzed each country individually, unadjusted associations were notably smaller than the pooled value used in the meta-analysis and very few countries showed significant associations between IPV and HIV (see Table 3 in [2]). By not allowing for this potential within-study heterogeneity, the already-substantial heterogeneity in results reported in the authors’ Figures 2 and 3 is likely to represent a lower bound.

Second, the conduct of meta-analyses using non-randomized data – particularly in the context of an association that may vary by time and place – is always subject to the risk of confounding by either measured or unmeasured factors [5, 6]. Meta-analysis aims to combine experimental data from several small but comparable studies with similar scope, and homogenous designs and patient populations, to achieve more precise effect estimates [7]. Ideally, meta-analyses use RCT data to avoid confounding and bias. Since this meta-analysis used observational data, and given the fact that we do not believe that individuals are randomly exposed to IPV or HIV, there is a strong likelihood that any unadjusted analyses of this relationship are confounded. Although Li and colleagues evaluated how well each included study identified and adjusted for confounding in their study, they themselves did not then adjust for such confounding in their meta-analysis, thus re-opening the possibility of confounding in their results. Indeed, as we show (again, in Table 3 of [2]), adjusting for conceptually precedent, individual-level covariates leaves no significant association between IPV and HIV in any of the countries we study.

The Cochrane Handbook for Systematic Reviews of Interventions notes that a systematic review need not include a meta-analysis and “[i]f there is considerable variation in results, and particularly if there is inconsistency in the direction of effect, it may be misleading to quote an average value for the intervention effect.” (Section 9.5.3 of [8]). We believe that this paper highlights the potential hazards of using aggregate data to conduct meta-analysis of observational data, particularly when the data extracted from published papers ares not adjusted for possible confounding variables. Even when literature is identified through an exhaustive systematic review, it may not be feasible or responsible to conduct a meta-analysis if the identified papers are not deemed comparable, or heterogeneity across trials and populations is detected [3]. Should the authors still wish to conduct an analysis, alternative approaches, such as using fully individual-level data [9], meta-regressions, or a combination of aggregated and individualized data [10], may also prove fruitful in generating trustworthy effect measures.
